# Occurrence of Lipophilic Marine Toxins in Shellfish from Galicia (NW of Spain) and Synergies among Them

**DOI:** 10.3390/md13041666

**Published:** 2015-03-25

**Authors:** Laura P. Rodríguez, Virginia González, Aníbal Martínez, Beatriz Paz, Jorge Lago, Victoria Cordeiro, Lucía Blanco, Juan Manuel Vieites, Ana G. Cabado

**Affiliations:** ANFACO-CECOPESCA, Carretera del Colegio Universitario 16, 36310 Vigo PO, Spain; E-Mails: lperez@anfaco.es (L.P.R.); Virginia@anfaco.es (V.G.); anibal@anfaco.es (A.M.); beapaz@anfaco.es (B.P.); jlago@anfaco.es (J.L.); victoria__bibi@hotmail.com (V.C.); lucia@anfaco.es (L.B.); jvieites@anfaco.es (J.M.V.)

**Keywords:** synergies, lipophilic toxins, LC–MS/MS, cell culture, neuroblastoma

## Abstract

Lipophilic marine toxins pose a serious threat for consumers and an enormous economic problem for shellfish producers. Synergistic interaction among toxins may play an important role in the toxicity of shellfish and consequently in human intoxications. In order to study the toxic profile of molluscs, sampled during toxic episodes occurring in different locations in Galicia in 2014, shellfish were analyzed by liquid chromatography tandem mass spectrometry (LC–MS/MS), the official method for the detection of lipophilic toxins. The performance of this procedure was demonstrated to be fit for purpose and was validated in house following European guidelines. The vast majority of toxins present in shellfish belonged to the okadaic acid (OA) group and some samples from a particular area contained yessotoxin (YTX). Since these toxins occur very often with other lipophilic toxins, we evaluated the potential interactions among them. A human neuroblastoma cell line was used to study the possible synergies of OA with other lipophilic toxins. Results show that combination of OA with dinophysistoxin 2 (DTX2) or YTX enhances the toxicity triggered by OA, decreasing cell viability and cell proliferation, depending on the toxin concentration and incubation time. The effects of other lipophilic toxins as 13-desmethyl Spirolide C were also evaluated *in vitro*.

## 1. Introduction

Toxic episodes are originated by harmful microalgae that produce marine biotoxins. These compounds constitute, at present, the most important challenge for shellfish harvesting and marketing. Global production of mussels was 1,901,313 tonnes during 2010, of which 476,656 tonnes was from European aquaculture [1]. Galicia, in the North West of Spain is the most important European producer, with more than 189,000 tonnes in 2010 (although other sources point up to 300,000 tonnes) [2,3]. Galicia has been suffering harmful algal blooms (HAB) episodes since the mid-seventies [4]. The frequency and duration of these episodes have been increasing since then, and whether a plateau has been reached or not is a matter of concern [3]. This increase has been proposed to be related to an increase in the renewal time of the embayments, associated with global warming [5], although other possible causes have been proposed such us eutrophication, the increase of aquaculture or an improved scientific awareness [6].

Red tides are considered as one of the major environmental factors affecting Galician bivalve’s aquaculture [3]. From an economic point of view, HABs affect not only producers, but also depuration facilities and transforming companies. Indeed, taking into account that mussel production accounts for 98% of Galician bivalve production (in tons) and 81% (in €) [7], we can establish that mussels are one of the species most influenced by toxins in Galicia, since during many months in the year shellfish harvesting is forbidden in some producing areas due to the presence of toxins. Depending on the severity of the toxic episode, shellfish collection has been prohibited up to 80% of the year in some producing areas [3]. In the North West of Spain, diarrhetic shellfish poisoning (DSP) episodes are the most common and the most reported [6,8,9]. We have focused on lipophilic marine biotoxins that are currently regulated by European Union (EU) legislation, namely okadaic acid (OA) and analogues, the azaspiracid (AZA)-group toxins, the yessotoxin (YTX)-group toxins and the pectenotoxin (PTX)-group toxins. Among them, the okadaic acid toxin group is the most important lipophilic toxin from both quantity and frequency point of view [8,9,10,11]. Nevertheless, other lipophilic toxins have been isolated in Galicia: PTXs [9,11,12], YTXs [9,13], AZAs [14], and 13-desmethyl spirolide C (SPX-1) [9,11,15].

The symptoms of diarrhetic shellfish poisoning (DSP) include gastrointestinal complications such as diarrhea, nausea, vomiting or abdominal pain [16], that usually start between 30 min to a few hours after the ingestion and disappear within three days [17]. Toxins involved in DSP are okadaic acid (OA) and its analogues, the dinophysistoxins (DTXs): DTX1, DTX2, and DTX3 [18,19].

OA was isolated for the first time from the marine sponge of the genus *Halichondria* [20]. These toxins were subsequently shown to be produced by marine dinoflagellates belonging to the *Dinophysis* and *Prorocentrum* genera [18,21], which were isolated in Galician waters and are controlled in rutinary HAB monitoring [6,12,22,23,24].

**Figure 1:**
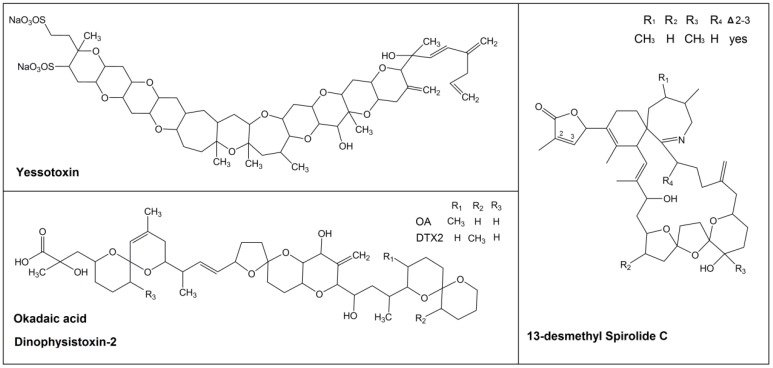
Chemical structures of yessotoxin (YTX), okadaic acid (OA), dinophysistoxin-2 (DTX2) and 13-desmethyl spirolide C (SPX1).

DTX1 is the dominant toxin in Japan, Canada, and Norway [25,26,27], while OA is the predominant toxin in Europe. Moreover DTX2, detected for the first time in Ireland during the 90s [28], has been detected in Spain and Portugal [11,12,23,29,30,31,32,33]. In addition, it is known that toxins of the OA group are accompanied sometimes by other lipophilic toxins, such as YTXs, cyclic imines (CIs), PTXs or AZAs [9,12,13,14,15]. The chemical structures of the parent compound of some of these groups of toxins are shown in Figure 1.

Yessotoxins were isolated for the first time in 1986 in Japan from the digestive glands of the scallop *Patinopecten yessoensis* [34]. This polycyclic ether compound is produced by the dinoflagellates *Protoceratium reticulatum* [35], *Lingulodinium polyedrum* [36], and *Gonyaulax spinifera* [37]. Yessotoxins were first detected in Galicia in 2006 [13] and YTX producing *Lingulodinium polyedrum* and *Protoceratium reticulatum* strains were isolated [38,39]. YTXs were first included within the DSP group due to their occurrence with DTX1 and DTX3 [40], but nowadays YTXs are independently regulated in the EU legislation [41,42] since it was demonstrated that these compounds display different mechanisms of action [43].

On the other hand, cyclic imines are a group of macrocyclic compounds with a cyclic imine moiety in their chemical structure. Spirolides, gymnodimines, pinnatoxins, pteriatoxins, prorocentrolides, and spiro-prorocentrimine belong to the CI group [44,45,46,47,48,49]. The largest group of CIs is the spirolide (SPX) group, of which 13-desmethyl spirolide C (SPX1) is the most common analogue found in shellfish. Spirolides were discovered in Nova Scotia (Canada) in the early 1990s during the routine monitoring of DSP toxins [50]. These compounds are produced by the dinoflagellates *Alexandrium ostenfeldii* and *Alexandrium peruvianum* [51,52,53]. As far as we know, references reporting SPX in Galicia are scarce [9,11,15] and they are related to just two different toxic events. The first event was in 2005 [11,15]. Regarding the third reference, this is focused on methodology validation and the available information regarding samples is that they were collected between 2010–2011 [9]. Taking into account that SPX are not currently legislated, it is difficult to know if SPX is uncommon in Galician waters or if it is not commonly analyzed.

The European Union (EU) has set a regulatory limit of 160 µg/kg for the sum of OA, DTXs, and pectenotoxins; 160 µg/kg for AZAs [41] and 3.75 mg/kg for YTX [42]. On the contrary, no limits have been set for any compounds belonging to the CI group [54].

The global aim of this work was to evaluate the presence of lipophilic marine toxins in shellfish harvested from Galicia and their effects on human cells. On one hand, the occurrence of these toxins in shellfish during a fixed period of time was studied. On the other hand, the potential effects of different toxins found together in the same mollusc were assessed. Both goals represent a real scenario where consumption of bivalves containing several types of lipophilic toxins may occur.

## 2. Results and Discussion

### 2.1. Lipophilic Toxins Analysis

With the aim to characterize the lipophilic toxin profiles of shellfish farmed in Galicia, a complete analysis of the toxin profiles was carried out using the LC–MS/MS technique. Bivalve samples were collected and analyzed during September–November 2014 from both open and closed harvesting areas.

We analyzed all the lipophilic toxins currently legislated in the EU: OA group toxins, PTXs group toxins, YTXs group toxins, and AZAs group toxins [41]. The limits of quantitation (LOQ), based on the lowest point of the respective calibration curve for each group of toxins are the following: 40 μg eq OA/kg for OA, DTX2, DTX3, PTXs; 40 μg eq AZA/kg for AZAs, and 60 μg eq YTX/kg for yessotoxins.

**Table 1:** Presence and concentration of lipophilic toxins in raw molluscs from Galician Rias. OA: okadaic acid; DTX2: dinophysistoxin-2; YTX: yessotoxin; PTX: pectenotoxin; LOQ: limit of quantitation.

Matrix	Free OA (μg eq OA/kg)	Total OA (μg eq OA/kg)	Free DTX2 (μg eq OA/kg)	Total DTX2 (μg eq OA/kg)	YTX (mg eq YTX/kg)	45-OH-YTX (mg eq YTX/kg)	Total YTX (mg eq YTX/kg)	PTX-2 (μg eq AO/kg)
*M. galloprovincialis*	64	108	<LOQ	<LOQ	<LOQ	<LOQ	<LOQ	<LOQ
*M. galloprovincialis*	85	128	<LOQ	<LOQ	<LOQ	<LOQ	<LOQ	<LOQ
*M. galloprovincialis*	98	129	<LOQ	<LOQ	<LOQ	<LOQ	<LOQ	<LOQ
*M. galloprovincialis*	36	79	<LOQ	<LOQ	<LOQ	<LOQ	<LOQ	<LOQ
*M. galloprovincialis*	46	129	<LOQ	<LOQ	<LOQ	<LOQ	<LOQ	<LOQ
*M. galloprovincialis*	52	85	<LOQ	<LOQ	<LOQ	<LOQ	<LOQ	<LOQ
*M. galloprovincialis*	73	118	<LOQ	<LOQ	<LOQ	<LOQ	<LOQ	<LOQ
*M. galloprovincialis*	128	142	<LOQ	<LOQ	<LOQ	<LOQ	<LOQ	<LOQ
*M. galloprovincialis*	47	135	<LOQ	<LOQ	<LOQ	<LOQ	<LOQ	<LOQ
*M. galloprovincialis*	46	128	<LOQ	<LOQ	<LOQ	<LOQ	<LOQ	<LOQ
*M. galloprovincialis*	56	147	<LOQ	<LOQ	<LOQ	<LOQ	<LOQ	<LOQ
*M. galloprovincialis*	86	162	<LOQ	<LOQ	<LOQ	<LOQ	<LOQ	<LOQ
*M. galloprovincialis*	55	92	<LOQ	<LOQ	<LOQ	<LOQ	<LOQ	<LOQ
*M. galloprovincialis*	26	78	<LOQ	<LOQ	<LOQ	<LOQ	<LOQ	<LOQ
*M. galloprovincialis*	41	180	<LOQ	<LOQ	<LOQ	<LOQ	<LOQ	<LOQ
*M. galloprovincialis*	76	201	<LOQ	<LOQ	<LOQ	<LOQ	<LOQ	<LOQ
*M. galloprovincialis*	261	361	<LOQ	<LOQ	<LOQ	<LOQ	<LOQ	<LOQ
*M. galloprovincialis*	46	103	<LOQ	<LOQ	<LOQ	<LOQ	<LOQ	<LOQ
*M. galloprovincialis*	87	193	<LOQ	<LOQ	<LOQ	<LOQ	<LOQ	<LOQ
*M. galloprovincialis*	76	162	<LOQ	<LOQ	<LOQ	<LOQ	<LOQ	<LOQ
*M. galloprovincialis*	169	376	<LOQ	<LOQ	<LOQ	<LOQ	<LOQ	<LOQ
*M. galloprovincialis*	69	107	<LOQ	<LOQ	<LOQ	<LOQ	<LOQ	<LOQ
*M. galloprovincialis*	42	83	<LOQ	<LOQ	<LOQ	<LOQ	<LOQ	<LOQ
*M. galloprovincialis*	<LOQ	<LOQ	<LOQ	<LOQ	<LOQ	<LOQ	<LOQ	<LOQ
*M. galloprovincialis*	<LOQ	<LOQ	<LOQ	<LOQ	<LOQ	<LOQ	<LOQ	<LOQ
*M. galloprovincialis*	<LOQ	44	<LOQ	<LOQ	<LOQ	<LOQ	<LOQ	<LOQ
*M. galloprovincialis*	96	177	<LOQ	<LOQ	<LOQ	<LOQ	<LOQ	<LOQ
*M. galloprovincialis*	41	59	<LOQ	<LOQ	<LOQ	<LOQ	<LOQ	<LOQ
*M. galloprovincialis*	42	67	<LOQ	<LOQ	<LOQ	<LOQ	<LOQ	<LOQ
*M. galloprovincialis*	29	64	<LOQ	<LOQ	<LOQ	<LOQ	<LOQ	<LOQ
*M. galloprovincialis*	52	78	<LOQ	<LOQ	<LOQ	<LOQ	<LOQ	<LOQ
*M. galloprovincialis*	43	104	<LOQ	<LOQ	<LOQ	<LOQ	<LOQ	<LOQ
*M. galloprovincialis*	157	259	<LOQ	<LOQ	<LOQ	<LOQ	<LOQ	<LOQ
*M. galloprovincialis*	73	135	<LOQ	<LOQ	0.49	<LOQ	0.49	<LOQ
*M. galloprovincialis*	80	183	<LOQ	<LOQ	<LOQ	<LOQ	<LOQ	<LOQ
*M. galloprovincialis*	<LOQ	64	<LOQ	<LOQ	0.25	<LOQ	0.25	<LOQ
*M. galloprovincialis*	<LOQ	<LOQ	<LOQ	<LOQ	0.59	0.11	0.7	<LOQ
*M. galloprovincialis*	<LOQ	<LOQ	<LOQ	<LOQ	0.3	0.08	0.38	<LOQ
*M. galloprovincialis*	<LOQ	43	<LOQ	<LOQ	0.19	<LOQ	0.19	<LOQ
*M. galloprovincialis*	<LOQ	56	<LOQ	<LOQ	0.21	<LOQ	0.21	<LOQ
*M. galloprovincialis*	<LOQ	<LOQ	<LOQ	<LOQ	0.11	<LOQ	0.11	<LOQ
*M. galloprovincialis*	<LOQ	<LOQ	<LOQ	<LOQ	0.12	<LOQ	0.12	<LOQ
*M. galloprovincialis*	<LOQ	<LOQ	<LOQ	<LOQ	0.27	0.08	0.35	<LOQ
*M. galloprovincialis*	<LOQ	<LOQ	<LOQ	<LOQ	0.09	<LOQ	0.09	<LOQ
*M. galloprovincialis*	<LOQ	<LOQ	<LOQ	<LOQ	0.15	<LOQ	0.15	<LOQ
*M. galloprovincialis*	<LOQ	42	<LOQ	<LOQ	0.24	<LOQ	0.24	<LOQ
*M. galloprovincialis*	<LOQ	<LOQ	<LOQ	<LOQ	0.08	<LOQ	0.08	<LOQ
*M. galloprovincialis*	<LOQ	<LOQ	<LOQ	<LOQ	0.14	<LOQ	0.14	<LOQ
*M. galloprovincialis*	<LOQ	<LOQ	<LOQ	<LOQ	0.16	<LOQ	0.16	<LOQ
*M. galloprovincialis*	<LOQ	<LOQ	<LOQ	<LOQ	0.1	<LOQ	0.1	<LOQ
*M. galloprovincialis*	<LOQ	<LOQ	<LOQ	<LOQ	0.09	<LOQ	0.09	<LOQ
*M. galloprovincialis*	<LOQ	<LOQ	<LOQ	<LOQ	0.09	<LOQ	0.09	<LOQ
*M. galloprovincialis*	<LOQ	<LOQ	<LOQ	<LOQ	0.15	<LOQ	0.15	<LOQ
*M. galloprovincialis*	<LOQ	<LOQ	<LOQ	<LOQ	0.2	<LOQ	0.2	<LOQ
*M. galloprovincialis*	<LOQ	<LOQ	<LOQ	<LOQ	0.18	<LOQ	0.18	<LOQ
*M. galloprovincialis*	<LOQ	<LOQ	<LOQ	<LOQ	0.13	<LOQ	0.13	<LOQ
*M. galloprovincialis*	<LOQ	<LOQ	<LOQ	<LOQ	0.23	<LOQ	0.23	<LOQ
*M. galloprovincialis*	<LOQ	<LOQ	<LOQ	<LOQ	0.17	<LOQ	0.17	<LOQ
*M. galloprovincialis*	63	105	<LOQ	<LOQ	<LOQ	<LOQ	<LOQ	<LOQ
*M. galloprovincialis*	<LOQ	<LOQ	<LOQ	<LOQ	<LOQ	<LOQ	<LOQ	<LOQ
*M. galloprovincialis*	697	890	87	92	<LOQ	<LOQ	0.45	<LOQ
*M. galloprovincialis*	84	129	<LOQ	<LOQ	0.40	0.07	<LOQ	46
*Venerupis spp*	<LOQ	<LOQ	<LOQ	<LOQ	<LOQ	<LOQ	<LOQ	<LOQ
*Chlamys varia*	<LOQ	391	<LOQ	<LOQ	<LOQ	<LOQ	<LOQ	<LOQ
*Chlamys varia*	<LOQ	104	<LOQ	83	<LOQ	<LOQ	<LOQ	<LOQ
*Chlamys varia*	<LOQ	100	<LOQ	185	<LOQ	<LOQ	<LOQ	<LOQ
*Chlamys opercularis*	<LOQ	101	<LOQ	<LOQ	<LOQ	<LOQ	<LOQ	<LOQ
*Pecten maximus*	<LOQ	<LOQ	<LOQ	<LOQ	<LOQ	<LOQ	<LOQ	<LOQ

The obtained results (Table 1) show that OA, either free or esterified, is the main toxin contaminating Galician molluscs, in accordance with previous reports [9], demonstrating that, as described previously, OA is the predominant toxin in Europe [4]. DTX2 was also found in a very low percentage of samples, but in a considerable concentration, at least half of the regulated limit, according to authors who had detected high amounts of DTX2 in Galicia and Portugal [9,29,30]. Nevertheless, *D. acuminata* is more frequent in Galician waters than *D. acuta* and is the causative agent of most harvesting area closures in Galicia due to DSP [55,56]. On the other hand, DTX2 is produced by *D. acuta*, which typically appears in autumn on the Galician coasts [57], although not every year. We know that *D. acuta* was present in this episode in low counts and that is the reason why DTX2 is scarcely detected in these samples.

In contrast, neither DTX1 nor AZAs analogues were detected in any of the samples studied, while YTX or its analogues were identified in a high percentage of the samples, although in very low concentrations. It is worth mentioning that those samples containing YTXs group toxins came from the same common restricted geographical origin (Sada, in Ría de Ares Betanzos), which was the provenance of the first reported YTX containing mussels case in Galicia [13]. In this location, YTX producing strains of *Lingulodinium polyedrum* had been previously isolated [38], although YTX producing species have been isolated in other places in Galicia [39].

Finally, we found a sample that presented a very low concentration of pectenotoxin-2 (PTX-2), a toxin that was previously detected in phytoplankton from Galician Rías [9,57]. Since PTX2 is synthesized by *D. acuta* and *D. caudata*, but not by *D. acuminata*, and this event was caused mainly by *D. Acuminata*, the low number of PTX2 positive samples found is easily explained. In fact, PTX-group toxins frequently co-occur with OA-group toxins, which appears to be the basis for grouping these toxins together in European regulations. Currently, PTXs are included in the regulatory limit for OA and analogues although they do not share the same mechanism of action [19].

### 2.2. Effects of Toxins on Cell Viability

In order to study the combined effect of lipophilic toxins that can be present together in the same mollusc and then, be responsible for seafood poisoning in humans, we first tested the loss of cell viability on a neuroblastoma cell line, after exposure to these compounds. We evaluated the toxic effects of different marine biotoxins and other toxic compounds on this cell line in previous studies [58,59,60,61,62].

**Figure 2:**
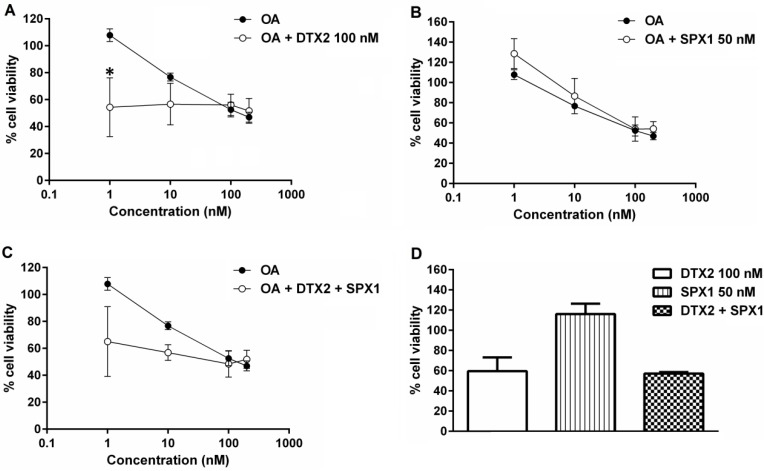
Effect of OA, DTX2 and SPX1 on neuroblastoma cell viability. Neuroblastoma cells were incubated for 48 h with 1–200 nM OA combined with 100 nM DTX2 (**A**); 50 nM of SPX1 (**B**) and both compounds (**C**); (**D**) Individual effects of DTX2 100 nM, SPX1 50 nM, and both compounds on neuroblastoma BE(2)-M17 cell line. Results are the mean ± SEM of two experiments performed in duplicate, and are expressed as percentage of fluorimetric values obtained with respect to the controls (untreated cells). * Denotes statistical significance.

For this purpose neuroblastoma cells were incubated for 48 h with OA (1–200 nM) combined with 100 nM of DTX2 (Figure 2A), 50 nM of SPX1 (Figure 2B) or a combination of both (Figure 2C). On the one hand, 100 nM DTX2 increased in a significant way the toxic potential of OA at the lowest concentration tested (1 nM) (Figure 2A). On the other hand, 50 nM SPX1 did not affect the dose response curve of OA on neuroblastoma cells viability (Figure 2B). As expected, the combined effect caused by DTX2 together with SPX1 on the OA toxicity, is similar to that triggered only by DTX2; and, it was confirmed that SPX1 is not toxic at the concentration used in these experiments. The individual effect of these toxins is illustrated in Figure 2D proving that DTX2, but not SPX1, decreases the neuroblastoma cell viability.

As mentioned before, OA and its analogues, DTX1, DTX2, and DTX3, form the group of OA-toxins. While OA and DTX2 differ in the position of one methyl group in the molecule, DTX1 presents one additional methyl group, and DTX3 represents a wide range of derivatives of OA—DTX1 and DTX2 esterified with saturated and unsaturated fatty acids. Based on LD_50_ experiments and following intraperitoneal injection in mice, the following toxic equivalence factors (TEFs) were established: OA = 1, DTX1 = 1, DTX2 = 0.6. For DTX3 the TEF values are equal to those of the corresponding unesterified toxins (OA, DTX1, and DTX2). It is well known that the mechanism of action of OA-group toxins consists of the inhibition of serine/threonine phosphoprotein phosphatases [19]. Although many studies concerning the toxicity of OA have been published [63], there is a lack of information related to DTXs. Even more, as far as we know, there are no reports based on the combined effects of these toxins from the same group. Indeed, the expert Panel of EFSA has presumed that the combined exposure to two or more toxins is additive with respect to dose (dose-addition), although data supporting this are currently lacking [19]. Therefore, in this study, and due to the fact that both compounds could be found in the same matrix, we combined OA and DTX2 with the aim to further investigate their relative toxic potency and the synergies between both types of toxins. In our hands, low concentrations of OA (1 nM) combined with DTX2, increase in a significant way the toxicity triggered by OA on neuroblastoma cells. Taking into account the results obtained in this study, we cannot indicate if DTX2 is less or more potent than OA, and this question has to be subsequently studied. In fact, a recent article mentions that DTX2 and OA show equal protein phosphatase inhibition activity [64].

Cyclic imines, including SPX1, were first discovered in the nineties, but at present they are not regulated in Europe or in other regions of the world. The toxicological data for these toxins is limited, comprising mostly acute toxicity studies [63]. They have not been categorically linked to human intoxication, although it was described that they act as potent antagonists on nicotinic acetylcholine receptors [65]. Nevertheless, there are not many reports considering the *in vitro* effects of these toxins on human cells. Based on the scarce bibliography, we used a 50 nanomolar concentration of SPX1. Under our conditions, we did not find any significant effect on neuroblastoma cells. Furthermore, this toxin did not modify the toxicity caused by DTX2 and OA. These studies confirm previous reports showing that SPX1 does not display cytotoxicity in a neuroblastoma cell line at the concentration tested (100 nM). These authors could not evaluate higher concentrations of this compound due to lack of standard of this toxin [66].

**Figure 3:**
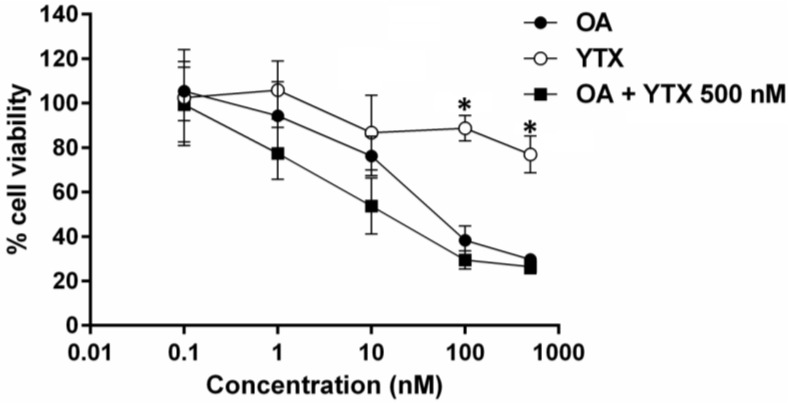
Effect of OA and YTX treatment on cell viability. Neuroblastoma cells were incubated for 48 h with OA YTX (1–500 nM), and OA combined with 500 nM YTX. Results are the mean ± SEM of two experiments performed in duplicate, and are expressed as percentage of fluorimetric values obtained with respect to controls (untreated cells). * Denotes statistical significance.

The group of YTXs consists of polyether compounds, with 11 contiguously transfused ether rings, an unsaturated side chain, and two sulfate esters. These toxins have already been identified in shellfish harvested from a particular area in Galicia [13]. Then, we evaluated the *in vitro* potential effect of YTX on a neuroblastoma cell line. Cells were incubated for 48 h with OA or YTX (1–500 nM) and with both together, 1–500 nM OA combined with 500 nM of YTX. As expected, YTX was less toxic than OA confirming previous studies reporting low toxicity of YTX in comparison with OA [59]. The specific dose-response curves show the highest significant differences at 100 and 500 nM concentrations. When both toxins were added together, no statistically significant differences were observed in the viability of neuroblastoma cells after exposure to OA (1–500 nM) in combination with 500 nM YTX (Figure 3).

### 2.3. Effect of Okadaic Acid and Yessotoxin on Cell Morphology

**Figure 4:**
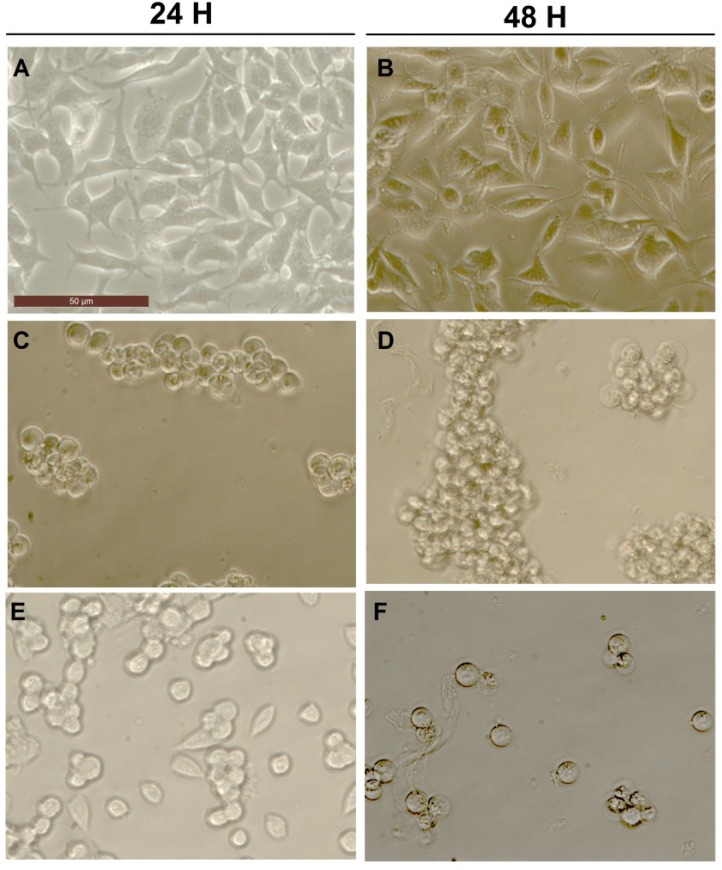
Photographs of BE(2)-M17 cell line incubated with OA and OA combined with YTX. Control cultures after 24 h (**A**) and 48 h incubation (**B**); Cells exposed 24 or 48 h to 500 nM OA (**C**,**D**) and 10 nM OA combined with 500 nM YTX (**E**,**F**).

After incubation for 24 or 48 h, OA induced cell damage and substrate detachment on neuroblastoma cells as detected on microscopic observation (Figure 4). This effect was previously reported, since OA induced apoptosis in neuroblastoma cells involving, among other effects, the disruption of F-actin cytoskeleton, the activation of caspase-3 and the collapse of the mitochondrial membrane potential [67]. Incubation with 500 nM OA for 24 h caused the complete loss of cell morphology and detachment (Figure 4C), with no differences after 48 h incubation (Figure 4D). This effect was time and dose dependent from 10 nM 24 h, (not shown). The combination of 500 nM YTX did not modify the effect of 10 nM OA after 24 h incubation (Figure 4E) or 48 h (Figure 4F).

### 2.4. Effects of Okadaic Acid and Yessotoxin on Cell Proliferation

**Figure 5:**
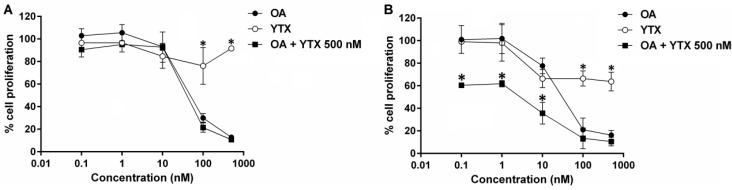
Effect of OA and YTX treatment on cell proliferation. Neuroblastoma cells were incubated for 24 h (**A**) or 48 h (**B**) with 1–500 nM OA combined with 500 nM of YTX (mean ± SEM; *n* = 2). Results are expressed as the percentage of fluorimetric values obtained with respect to controls (untreated cells). * Denotes statistical significance.

The effect of OA, and YTX and their combination on cell proliferation was tested in BE(2)-M17 cells exposed to these toxins for 24 and 48 h. Incubation with OA (0.1–500 nM) for 24 h induced a dose-dependent negative effect on cell proliferation. The addition of 500 nm YTX did not modify the OA dose-response after 24 h (Figure 5A). On the contrary, after 48 h incubation, the toxic potency of OA was significantly increased in combination with YTX, showing a very potent toxic effect at the lowest concentrations tested (0.1, 1, and 10 nM OA) (Figure 5B). These results are in good agreement with the observed effects on cell morphology previously described for both compounds (Figure 4E,F).

The toxicological information for YTX-group toxins is limited and includes mostly studies on their acute toxicity in mice, since reports on adverse effects in humans are absent. Regarding the mechanism of action of YTXs, four major molecular processes have been proposed, comprising the modulation of calcium movements among different cellular compartments, the modification of cellular cAMP levels, the alteration of protein disposal, and apoptosis. In addition, it was shown that YTX at nanomolar concentrations inhibits the beating frequency in cardiomyocytes *in vitro* [63,68]. Nevertheless, taking into account that YTXs can be present in the bivalves together with other lipophilic toxins, a study of the possible interactions among these toxins is desirable. In this sense, there is a lack of reports related to the potential synergic effects of YTX. In this study we proved that nanomolar concentrations of YTX by itself decrease the cell viability in accordance with previous studies, since YTX induces apoptotic events in different cellular lines [69]; although combination of YTX (500 nM) with OA did not increase the toxic effect of OA after short (24 h) incubation. Further research is needed in order to know if higher concentrations of YTX could promote the toxic potency of OA on cell viability. A similar effect is observed on cell proliferation, cell morphology, and cell detachment from the substrate. However, when a longer incubation is carried out in the presence of YTX (48 h), the toxicity of OA is statistically increased, mainly at the lower concentrations tested (0.1–10 nM). As far as we know, this is the first report proving the synergy between YTX and OA on different cellular parameters in a time and concentration dependent way.

In summary, in this study we confirmed the presence of OA, DTX2, YTX, 45-OH-YTX and PTX-2 in shellfish from the NW of Spain by using a LC–MS/MS method. The most prevalent toxin in this region was OA; other lipophilic toxins were found very occasionally, except for YTX and its analogues which were detected often but in a very specific area. Although the samples tested correspond to a relatively short time frame, these results correlate with previous work regarding predominance of OA [8,9,10,11]. When shellfish harvesting is allowed, concentrations of these toxins are low or even undetectable. However, during a toxic episode, levels increase and several toxins co-occur and can be present together in the same molluscs. Simulation of this scenario on an *in vitro* cellular model showed that combination of low levels of DTX2 or YTX with OA accentuates the toxic effect caused by OA alone increasing cellular damage or death, and hence, it emphasizes the interest of developing further studies on toxin interaction in an *in vivo* model.

## 3. Experimental Section

### 3.1. Reagents and Toxin Standards

The human neuroblastoma cell line BE(2)-M17 was purchased from the European Collection of Cell Cultures (Health Protection Agency, Salisbury, UK). OA, dinophysistoxin 2 (DTX2), YTX, homo YTX, PTX-2, AZA-1, AZA-2, AZA-3 and 13-desmethyl spirolide C (SPX-13) standard solutions were supplied by Laboratorio CIFGA S.A. (Lugo, Spain). OA (purity ≥ 98.9%), DTX2 (purity ≥ 98.9%), YTX (purity ≥ 97.9%), homoYTX (purity ≥ 98.4%), AZA-1 (purity ≥ 98%), AZA-2 (purity ≥ 97%) and AZA-3 (purity ≥ 96%) are Certified Reference Materials (CRMs). PTX-2 (purity ≥ 99%) is considered Quality Controlled Standard (QCS). The AlamarBlue^®^ Cell Viability Reagent and CyQUANT Cell Proliferation Assay Kit were purchased from Molecular Probes (Life Technologies Co., Carlsbad, CA, USA). HAM’s F-12 with stable glutamine medium, Eagle’s Minimum Essential Medium (EMEM), fetal bovine serum (FBS), gentamicin, amphotericin, non-essential amino acids (NEA), Phosphate Buffered Saline (PBS) and trypsin-EDTA solutions were from Sigma-Aldrich (Madrid, Spain). Costar 96-well assay plates with a clear bottom and tissue culture-treated surface were purchased from Corning Inc. (New York, NY, USA).

### 3.2. Sampling

**Figure 6:**
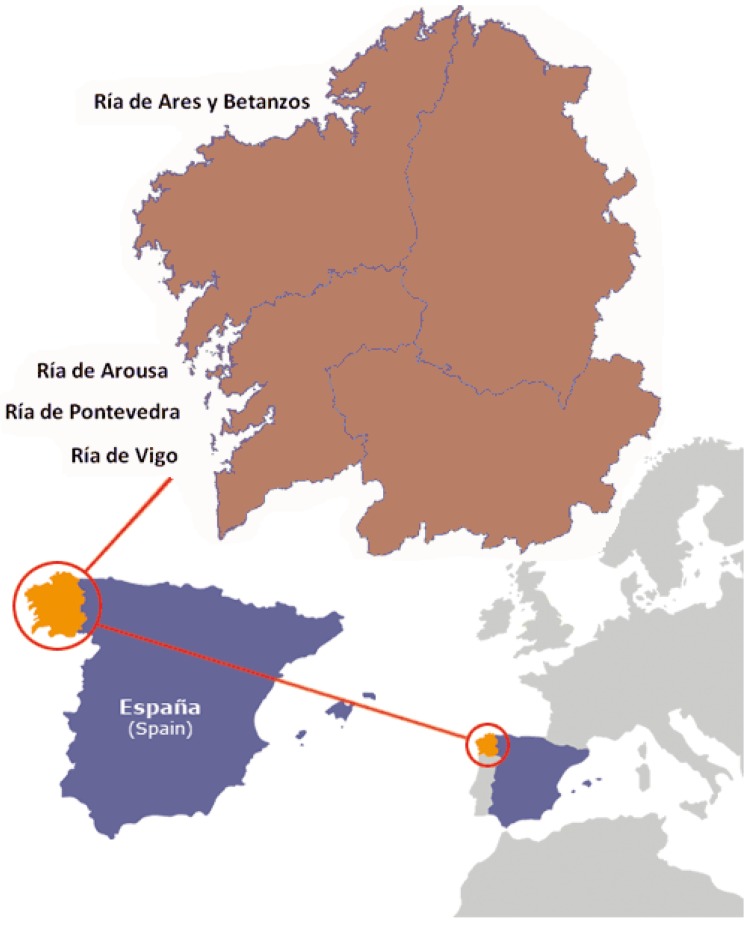
Sampling areas of the Galician Rias.

Mussels (*Mytilus galloprovincialis*), clams (*Venerupis* spp.), small scallops (*Chlamys varia*) and scallops (*Pecten maximus*) were collected through September–November 2014 from both open and closed harvesting areas. Samples were immediately processed after arrival in the laboratory or kept frozen until analyzed. Every sample was analyzed after homogenization of the whole sample. Marine biotoxin control in Galicia lies on the public organism INTECMAR (Technological Institute for the Control of Marine Environment in Galicia www.intecmar.org [70]), and on own-checks performed by producers and food business operators. INTECMAR allows or forbids molluscs harvesting in the different production areas and offers online information on the state of the production zones. Taking advantage of this information, mussels, clams, cockles, and scallops were collected by producers as usual, with the appropriate permission when necessary, from both open and closed areas. Figure 6 shows the different areas where molluscs were harvested.

### 3.3. Sample Extraction

Sample extraction was carried out following EU-Harmonised-SOP-LIPO-LCMSM-Version 5 [71]. Briefly, 100–150 g of shellfish flesh was pooled and homogenized with a blender and 2 g of whole body homogenate were extracted twice with 9 mL of methanol, using a tube shaker and a centrifugation step (2000× *g* for 10 min at 20 °C). Both extracts were pooled and adjusted to a final volume of 20 mL. An aliquot of the extract was submitted to alkaline hydrolysis by addition of NaOH 2.5 N at 76 °C for 40 min and finally neutralized with HCl 2.5 N in order to quantify the total content of OA/DTX toxins. Hydrolyzed and non-hydrolyzed extracts were filtered through a methanol-compatible 0.22 syringe filter before being injected in the LC–MS/MS equipment.

### 3.4. Liquid Chromatography–Mass Spectrometry (LC–MS/MS)

Lipophilic toxins analyses were performed by LC–MS/MS methodology using an UPLC chromatography system (Acquity H-class from Waters, Milford, MA, USA) coupled to a MS/MS detector (XEVO TQS from Waters) and a Waters Acquity UPLC BEH C18 1.7 µm 2.1 × 100 mm column. The analytical method was based on the EU-Harmonised-SOP-LIPO-LCMSM-Version 5 [71]. Analytical conditions are described in Table 2 and Table 3.

**Table 2:** LC-MS/MS conditions.

Column	Waters Acquity UPLC BEH C18 1.7 µm 2.1 × 100 mm	
Flow	0.40 mL/min	
Injection volume	2 μL	
Column temperature	45 °C	
Maximum pressure	15,000 psi	
Time (min)	% Mobile phase A	% Mobile phase B
	Ammonia 6.7 mM in water	Ammonia 6.7 mM in acetonitrile
initial	70	30
0.50	70	30
6.00	10	90
6.50	0	100
9.50	0	100
9.60	70	30
10.00	70	30

**Table 3:** MS/MS fragmentation.

Compound	Parent Ion (Da)	Fragment Ion (Da)	ESI	Cone Voltage (V)	Collision Voltage (eV)	Dwell (s)
AZA3 *	828.50	362.20	+	47	55	0.007
AZA3	828.50	640.40	+	47	55	0.007
AZA1	842.50	362.00	+	50	50	0.038
AZA1 *	842.50	654.50	+	50	55	0.036
AZA2 *	856.50	654.50	+	27	45	0.038
AZA2	856.50	672.50	+	27	45	0.038
PTX2 *	876.50	823.50	+	36	25	0.034
PTX2	876.50	841.50	+	36	20	0.034
PTX1	892.50	213.50	+	36	20	0.034
PTX1 *	892.50	821.50	+	36	25	0.034
PTX1	892.50	839.50	+	36	25	0.034
YTX	570.40	396.30	−	45	35	0.007
YTX *	570.40	467.40	−	45	30	0.007
h-YTX	577.50	403.40	−	48	30	0.007
h-YTX *	577.50	474.40	−	48	30	0.007
45-OH-YTX	578.40	396.40	−	75	35	0.007
45-OH-YTX *	578.40	467.40	−	75	30	0.007
45-OH-homo-YTX	585.50	403.40	−	75	30	0.007
45-OH-homo-YTX *	585.50	474.40	−	75	30	0.007
AO-DTX2	803.50	113.00	−	86	56	0.007
AO-DTX2 *	803.50	255.20	−	86	46	0.007
DTX1 *	817.50	255.20	−	86	48	0.007
DTX1	817.50	113.1	−	86	43	0.007

* Quantitative transitions.

The analytes were quantified with individual six-point external calibration curves prepared in methanol from reference standards. Calibration curve ranges were from 2–40 ng/mL for OA, DTX1, DTX2, PTX2, AZA1, AZA2 and AZA3; and from 6–120 ng/mL for YTX and homoYTX. Analyte concentration in tissue was calculated using peak area, sample weight, and toxicity equivalent factors (TEF) proposed on the EU Harmonised SOP, adopted by the European Food Safety Authority (EFSA) [71,72].

An in house validation was carried out following guidelines stated in Commission Decision 2002/657 [73], calculating selectivity/specificity, cedasticity, accuracy, precision, limit of detection (LOD), limit of quantitation (LOQ), robustness, linearity, and uncertainty.

Selectivity/specificity was based on retention time (RT) drift between samples and standard solutions. Confirmation of peaks was assessed by MS/MS fragmentations ratios for each toxin with a maximum tolerance ratio between quantitative and confirmative transitions, following the criteria for analytical methods described in Decision (EC) 2002/657 [73]. Standard solutions used were OA, DTX1, DTX2, PTX2, AZA1, AZA2, AZA3, YTX, and homoYTX. For PTX1, 45-OH-YTX and 45-OH-homoYTX (without standards available) we used the relative RT compared to PTX2, YTX and homo-YTX, respectively. In the case of OA and DTX2 with the same transitions at least a resolution of 1.0 was necessary for acceptable separation of peaks. Blank samples were analyzed to ensure the absence of interferences.

The accuracy of the method was estimated by calculating recoveries of toxins from spiked samples at different concentration levels. Study was done on different homogenized tissue matrices, such as raw mussels, oysters, or scallops. To estimate the precision, the assay was tested both under repeatability and intermediate precision conditions. The repeatability characteristics were estimated by analyzing several fractions of spiked samples. The intermediate precision of the test was estimated by analyzing the spiked matrices with toxin levels covering the working range of the assay on different days by the same analyst. These studies were performed at three levels: the LOQ, the regulatory limit and 1.5 times the regulatory limit. With the obtained data, the mean (x), the standard deviation (s), and the relative standard deviation or RSD (%RSD = s·100/x) were calculated. The RSD limit was calculated as ratio between the obtained RSD and the reference value RSD following the Horwitz or Horrat equations [74], which is dependent on the concentration level (C).

The limits of detection (LOD) and limits of quantitation (LOQ) in shellfish tissue for monitored toxins were 2 ng/mL on column and 40 μg eq/Kg for OA and AZA toxin groups; and 6 ng/mL on column and 60 μg eq/Kg for YTXs.

Linearity was determined from the six-point standard calibration curves analyzed before and after the analysis of a set of samples with the appropriate controls and blanks. A linear best fit was applied to each calibration curve (*R*^2^ > 0.98 in each case) and the deviation of the curve slopes between sample sets had to be lower than 25% to be considered as acceptable [71]. The uncertainty of the whole method at the interest level was estimated with the results obtained during validation, taking into account the precision of the method.

**Figure 7:**
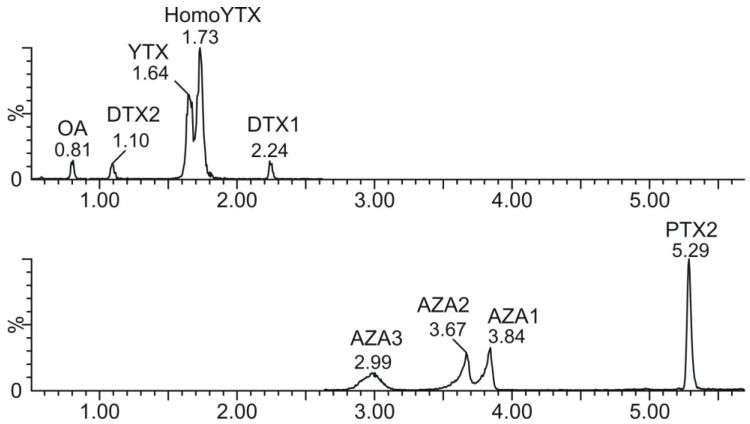
LC-MS/MS chromatograms for the standards of OA, DTX2, YTX, hYTX, DTX1, AZA3, AZA2, AZA1, and PTX2, in methanol:water (50:50), respectively.

**Figure 8:**
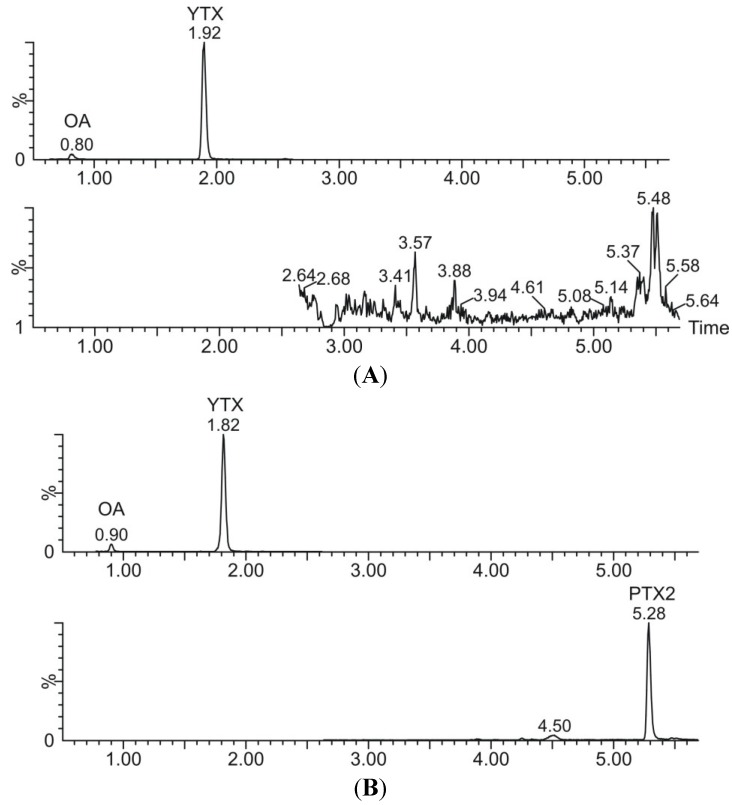
(**A**) Chromatogram of a hydrolyzed extract of a naturally contaminated mussel sample containing OA and YTX; (**B**) Chromatogram of the same naturally contaminated mussel sample from an unhydrolyzed extract containing OA, YTX, and PTX2.

An example of a chromatogram obtained with standards of different lipophilic toxins diluted in methanol:water (50:50), can be observed in Figure 7. A chromatogram of a naturally contaminated mussel is illustrated in Figure 8, from a hydrolyzed (Figure 8A) and a non-hydrolyzed (Figure 8B) extract.

### 3.5. Cell Culture

The BE(2)-M17 cell line (ATCC number CRL-2267) was grown in EMEM and Ham’s F12 medium (1:1), 1% NEA, 50 μg·mL^−1^ gentamicin, 50 ng·mL^−1^ amphotericin B and 10% FBS. Cells were grown in 25 cm^2^ tissue flasks incubated in a humidified atmosphere containing a constant level of 5% CO_2_ at 37 °C until 70%–90% confluence was reached. Cells were sub-cultured with fresh medium by first washing with PBS followed by release with a trypsin/EDTA solution twice per week. Cells were seeded onto 96-well plates at a density of 2500 cells/well 48 h prior to treatment to achieve 80% of cell confluence and subsequently used for cytotoxicity assays.

### 3.6. Cell Viability Assay

Cytotoxicity of the different lipophilic toxins was evaluated using the fluorescent probe Alamar Blue. This reagent changes due to cell metabolism from a non-fluorescent blue color to a reduced pink fluorescent form, when it is added to the culture medium. Briefly, BE(2)-M17 cells were seeded onto 96-well plates at a density of 2500 cells/well 48 h prior to treatment. After removing the medium, cells were exposed in duplicate to different concentrations of OA, DTX2, YTX, and SPX-13 in the culture medium. After 48 h treatment, the fluorescent reagent Alamar Blue was aseptically added to the wells and incubated for 3 h at 37 °C following the recommendations of the commercial source. Fluorescence was measured using a Spectra Max M5 microplate reader from Molecular Devices Inc. (Sunnyvale, CA, USA) at 560 nm excitation and 585 nm emission wavelength. Results are expressed as the percentage of the increment in the fluorescence of toxin-treated cells *vs* the increment of fluorescence in control wells (100% of viability).

### 3.7. Light Microscopy

Neuroblastoma cells seeded on plates and incubated with different concentrations of toxins were visualized with an Olympus optic microscope to observe the cell morphology and substrate detachment. Images were recorded with a Leica color digital camera (DFC 480).

### 3.8. Cell Proliferation Assay

A CyQUANT Cell Proliferation Assay Kit was used to quantify cell proliferation. After 24 or 48 h of incubation with OA and YTX, culture plates were centrifuged at 3000 rpm for 5 min. The supernatant was discarded, and plates were stored at −20 °C until analysis, to allow for efficient cell lysis. On the day of the assay, a 1:20 CyQUANT cell lysis buffer solution was prepared in Milli-Q water, and CyQUANT GR dye was diluted (1:400) in the buffer solution. Then, 200 μL of this preparation was added to each well, and microplates were incubated at room temperature and protected from light. After 2–5 min, the fluorescence intensity was measured by a Spectra Max M5 microplate reader, with excitation at 485 nm and emission at 530 nm.

### 3.9. Data Analysis

Results are expressed as mean ± standard error of the mean (SEM) of *n* experiments. For every experiment all conditions were tested in duplicate. The statistical significance was determined using a *t*-test for unpaired data and the ANOVA paired test with Tukey multiple comparison post-test was used in the cytotoxicity studies. *p* < 0.05 was considered for significance.
